# GV1001, hTERT Peptide Fragment, Prevents 5-Fluorouracil-Induced Mucositis by Inhibiting Mitochondrial Damages

**DOI:** 10.3390/cells15090774

**Published:** 2026-04-25

**Authors:** Cheyenne Beheshtian, Wei Chen, Seojin Kim, Angela Jun, Eun-Bin Bae, Reuben Kim, Sangjae Kim, No-Hee Park

**Affiliations:** 1 The Shapiro Family Laboratory of Viral Oncology and Aging Research, UCLA School of Dentistry, 714 Tiverton Ave, Los Angeles, CA 90095, USA; cbeheshtian@g.ucla.edu (C.B.); chenwei304@g.ucla.edu (W.C.); sj25kim@gmail.com (S.K.); ebbae@g.ucla.edu (E.-B.B.); rkim@dentistry.ucla.edu (R.K.); 2 Jonsson Comprehensive Cancer Center, University of California, Los Angeles, CA 90095, USA; 3 Teloid Inc., 920 Westholme Avenue, Los Angeles, CA 90024, USA; sjkim@teloid.com; 4 Department of Medicine, David Geffen School of Medicine at University of California, Los Angeles, 10833 Le Conte Ave, Los Angeles, CA 90095, USA

**Keywords:** GV1001, 5-flurouracil, mucositis, mitochondria, chemotherapy

## Abstract

**Highlights:**

**What are the main findings?**

- GV1001, a peptide fragment from human telomerase reverse transcriptase, significantly attenuated 5-fluorouracil (5-FU)-induced mucositis in the gastrointestinal tract of mice, which could be induced by mitochondrial damages caused by 5-FU.
- GV1001 preserved mitochondrial integrity and bioenergetic function by reducing mitochondrial oxidative stress via preferentially binding cardiolipin, thereby preventing mitochondrial dysfunction induced by chemotherapy.
- GV1001 selectively protects normal human epithelial cells without diminishing the 5-FU’s cytotoxicity in human cancer cells.

**What is the implication of the main findings?**

- These findings identify GV1001 would be useful for the prevention of chemotherapy-induced mucositis (CIM).

**Abstract:**

Chemotherapy-induced mucositis (CIM) is a dose-limiting toxicity of cancer therapy that is mainly associated with mitochondrial dysfunction in epithelial cells. We investigated whether GV1001, a mitochondrial protective peptide from human telomerase reverse transcriptase (hTERT), attenuates 5-fluorouracil (5-FU)-induced mucositis in a murine model. 5-FU induced notable mortality, leukopenia, and mucositis in the gastrointestinal (GI) tract, including tongue, esophagus and small intestine. It promoted epithelial–mesenchymal transition (EMT), nuclear factor kappa-B (NF-κB) activation, systemic and mucosal inflammation, DNA damage, impaired cell proliferation, and apoptosis throughout the GI tract. GV1001 blocked 5-FU–associated mortality, significantly attenuated leukopenia, and notably prevented mucositis. GV1001 also suppressed 5-FU-induced DNA damage, EMT, loss of proliferative capacity, apoptosis, and NF-κB activation in mucosal epithelium. In normal human keratinocytes, 5-FU inhibited the cell proliferation, disrupted mitochondrial function, as evidenced by reduced mitochondrial membrane potential, increased reactive oxygen species (ROS) production, impaired electron transport chain (ETC) complex integrity, decreased ATP synthesis, and cytochrome c release into the cytosol. GV1001 markedly mitigated these 5-FU-induced mitochondrial defects. Taken together, GV1001 mitigates CIM by most likely preserving mitochondrial integrity and function, supporting its potential as a strategy to prevent cancer chemotherapy-associated mucosal injury in patients.

## 1. Introduction

Chemotherapy-induced mucositis (CIM) is a multifactorial inflammatory injury of the epithelial cells of the gastrointestinal (GI) tract, which develops within days of treatment initiation [1]. The condition is driven by the excessive generation of reactive oxygen species (ROS), the induction of pro-inflammatory cytokines, and the activation of nuclear factor kappa-B (NF-κB)-dependent transcriptional programs, which together orchestrate a cascade of epithelial and submucosal damage [1]. Clinically, mucositis manifests as painful ulcerative lesions, affecting approximately 40% of patients receiving standard cycled chemotherapy regimens [1]. In severe cases, mucositis can result in significant loss of body mass, and when compounded by chemotherapy-induced neutropenia, markedly increases the risk of bacteremia and sepsis, which frequently necessitates dose reductions or interruption of therapy [2,3].

5-Fluorouracil (5-FU), a widely used chemotherapeutic agent for the treatment of gastrointestinal, colorectal, and head and neck cancers, is well known to induce GI mucositis. This condition represents a common and dose-limiting toxicity, affecting up to 50–80% of treated patients. It is characterized by a range of clinical manifestations, including weight loss, leukopenia, oral ulceration, intestinal atrophy, and diarrhea [4,5,6,7]. At the molecular level, 5-FU induces cytotoxicity by disrupting thymidylate synthesis, inhibiting DNA and RNA synthesis, producing replication stress, and impairing ribosome biogenesis [8]. Also, 5-FU induces mucositis by suppressing key respiratory chain genes, disrupts complex I assembly, and triggers excessive mitochondrial reactive oxygen species (mtROS) production in rodent animal models. This oxidative burst promotes cytochrome c release to the cytosol, caspase-9 activation, and crypt-villus apoptosis characteristic of mucositis [9,10,11]. 5-FU disrupts Complex I, II, and IV enzymatic activity, collapses the mitochondrial membrane potential (ΔΨ_m_), and markedly increases mtROS in murine intestine [12]. 5-FU also induces epithelial–mesenchymal transition (EMT) as evidenced by the acquisition of mesenchymal markers in oral and intestinal epithelia [13,14]. Moreover, this oxidative burst activates NF-κB signaling pathways [14]. Together, mitochondrial dysfunction, EMT, and NF-κB activation form interlocking circuits that drive 5-FU-induced mucosal injury.

GV1001, a 16-amino acid peptide originating from telomerase reverse transcriptase (hTERT), was initially developed as a cancer vaccine, but has emerged as a multifunctional agent with anti-inflammatory, antioxidant, and cytoprotective properties [15,16,17]. Accumulating evidence indicates that GV1001 exerts direct intracellular actions, particularly at the level of mitochondrial regulation. It has been shown to preserve mitochondrial integrity, resulting in the inhibition of oxidative stress and apoptosis under conditions of cellular injury [18]. It attenuates doxorubicin- and cisplatin-induced mtROS accumulation, stabilizes mitochondrial membrane potential, and maintains mitochondrial function. In parallel, GV1001 suppresses key inflammatory signaling pathways, including NF-κB activation, reduces pro-inflammatory cytokine production, and inhibits epithelial–mesenchymal transition (EMT), a process implicated in tissue injury and remodeling [15,18]. These findings suggest that GV1001 targets interconnected pathways linking mitochondrial damage, oxidative stress, and inflammation. Such mechanisms are highly relevant to the pathogenesis of chemotherapy-induced mucositis, particularly in the context of 5-FU treatment.

Thus, we investigated whether GV1001 mitigates 5-FU-induced mucosal injury, possibly through preservation of mitochondrial function, using complementary in vivo and in vitro models.

## 2. Materials and Methods

### 2.1. Cell Culture

Normal human oral keratinocytes (NHOK) and immortalized non-tumorigenic human oral keratinocytes-16B (HOK-16B), prepared as previously described [19,20], were cultured in EpiLife^TM^ Medium (Thermo Fisher Scientific, MEPI500CA, Waltham, MA, USA). Human oral squamous cell carcinoma line (SCC-17B/UM-SCC-17B; MilliporeSigma, #SCC075, St. Louis, MO, USA) was cultured with Dulbecco’s Modified Eagle’s Medium (DMEM; Invitrogen, #11995123, Carlsbad, CA, USA) containing 10% fetal bovine serum (FBS; Thermo Fisher Scientific, #10437028). All cell cultures were grown to approximately 80% confluence prior to experimental treatment.

### 2.2. Animal Experiments and Animal Welfare

Thirty 8-week-old male C57BL/6J mice (Jackson Laboratories, Bar Harbor, ME, USA) were housed under specific pathogen-free conditions with ad libitum access to food and water, as described previously [18]. Mice were acclimated for one week before study initiation and randomly assigned to four experimental groups. All injections were administered every 48 h for two weeks, with a volume of 0.1 mL per injection.

1. PBS control (*n* = 7): intravenous (i.v.) injection of sterile phosphate-buffered saline (PBS) and intraperitoneal (i.p.) injection of sterile PBS (PBS/PBS).
2. GV1001 alone (*n* = 7): i.v. PBS inj. and i.p. GV1001 (2 mg/kg) inj., a dose previously shown to exert anti-inflammatory effects without toxicity in murine models [18] (PBS/GV1001).
3. 5-FU alone (*n* = 8): i.v. 5-fluorouracil (5-FU; 50 mg/kg) inj. and i.p. PBS inj., a regimen established to induce antitumor activity and mucositis [7,21] (5-FU/PBS).
4. 5-FU + GV1001 (*n* = 8): i.v. 5-FU (50 mg/kg) inj. and i.p. GV1001 (2 mg/kg) (5-FU/GV1001) inj.

Animal health and behavior were checked daily, and body weight was recorded every other day throughout the study. On Day 14, mice were anesthetized with ketamine/xylazine anesthesia (100 and 5 mg/kg, respectively) and were then euthanized. The University of California, Los Angeles Animal Research Committee approved all procedures (ARC protocol #2019-057), which complied with U.S. Department of Agriculture Animal Welfare Act provisions. Sample sizes of 7–8 animals per group were chosen based on prior 5-FU mucositis studies demonstrating adequate statistical power to detect meaningful intergroup differences [7].

GV1001 was offered by GemVax/Kael, Inc., (Seongnam-si, Republic of Korea). 5-FU was obtained from MilliporeSigma (#F6627, Burlington, MA, USA). The experimental timeline is shown in Supplementary Figure S1.

### 2.3. Collection of Serum and Tissue

Under general anesthesia, whole blood was obtained by cardiac puncture. White blood cell (WBC) counts were determined using a hemocytometer-based method, as previously described [22]. In brief, peripheral blood samples were collected from mice and diluted with Türk’s solution (MilliporeSigma, #1.09277) for 30 min to ensure complete hemolysis of erythrocytes and adequate staining of leukocyte nuclei. The diluted samples were then loaded onto a hemocytometer, and the total WBCs were counted under a light microscope. WBCs were identified by their nuclei with dark purple staining. Blood samples were then spun at 2000× *g* for 15 min to isolate serum. Then, pro-inflammatory cytokines were measured through enzyme-linked immunosorbent assay (ELISA) using commercial kits for interleukin-1β (IL-1β; Thermo Fisher Scientific, #BMS6002), tumor necrosis factor-alpha (TNF-α; Thermo Fisher Scientific, #BMS607-3), and interleukin-6 (IL-6; Thermo Fisher Scientific, #BMS603), as previously described [15].

Then, the tongue, esophagus, and small intestine were excised from each animal. Excised tongues were immersed intact in 1% toluidine blue (TB; MilliporeSigma, #198161) prepared in 10% (*v*/*v*) acetic acid for 60 s at room temperature. Specimens were destained by three sequential 30-s washes in fresh 1% acetic acid, followed by a brief PBS rinse. The dorsal and lateral tongue surfaces were photographed under standardized lighting and camera settings, and ulcerated surface area was reported as a percentage of ulcerative area versus tongue surface. Esophagus, small intestine, and tongue specimens were fixed in 4% paraformaldehyde (PFA) for H&E and immunofluorescent staining.

### 2.4. Hematoxylin and Eosin (H&E) Staining

Paraffin-embedded esophageal, intestinal, and tongue specimens were cut into 5-μm sections and stained with H&E following a previously described protocol [23]. Epithelial thickness, defined as the distance from the basement membrane to the outermost apical surface, was quantified in each tissue type using ImageJ version 1.54 [24].

### 2.5. Immunofluorescence (IF) Imaging and Analysis

For tissue immunofluorescent (IF) staining, paraffin-embedded esophagus, intestine, and tongue sections were incubated with various primary antibodies against: TNF-α (Abcam, #ab6671, Cambridge, UK), IL-6 (Thermo Fisher Scientific, #701028), IL-1β (Santa Cruz Biotechnology, #SC-32294, Dallas, TX, USA), alpha smooth muscle actin (α-SMA; MilliporeSigma, #A2547), E-Cadherin (BD Biosciences, #610181, San Jose, CA, USA), phosphorylated NF-κB p65 subunit (p-p65; Cell Signaling, #3036, Danvers, MA, USA), proliferating cell nuclear antigen (PCNA; Santa Cruz Biotechnology, #sc-56, Santa Cruz, CA, USA), Tumor protein 63 (p63; Abcam, #ab238080), phosphorylated histone H2A.X (γ-H2AX; MilliporeSigma, #05-636), N-cadherin (Abcam, #ab98952), and anti-monocyte/macrophage (MOMA-2; Abcam, #ab33451). Secondary antibodies utilized for fluorometric detection were Alexa Fluor 488-conjugated secondary antibody (Thermo Fisher Scientific, #A11029) or Alexa Fluor 546-conjugated secondary antibody (Thermo Fisher Scientific, #A11010).

For immunocytochemistry (ICC) staining, cells were treated with PBS, 5-FU, and/or GV1001 for 48 h, cells were fixed with 4% PFA and then incubated with different primary antibodies against: Cytochrome c Oxidase Subunit IV (COX IV; Cell Signaling Technology, #4850, Danvers, MA, USA), NADH Dehydrogenase Fe-S Protein 1 (NDUFS1; Cell Signaling Technology, #70264), Succinate Dehydrogenase Complex Iron Sulfur B (SDHB; Cell Signaling Technology, #92649), Ubiquinol-Cytochrome c Reductase Rieske Iron-Sulfur Polypeptide 1 (UQCRFS1; Cell Signaling Technology, #95231), Cytochrome C (Invitrogen, #MA5-11674). Secondary antibodies utilized for fluorometric detection were Alexa Fluor 488- or 546-conjugated secondary antibodies.

For IF probe staining, prior to 4% PFA fixation, primary NHOK and HOK-16B were stained with the following probes: fluorescent intracellular ROS probe (MilliporeSigma, #MAK143), MitoSox^TM^ Red mtROS; Invitrogen, #M36008), Biotracker^TM^ Mitochondrial FerroGreen live cell probe (Mito-FerroGreen; MilliporeSigma, #SCT262), MitoTracker Red CMXRos (Cell Signaling Technology, #9082), Mitochondrial Lipid Peroxide Live Cell Ferroptosis Indicator (MitoPeDPP; EMD Millipore Corp., #SCT261, Burlington, MA, USA), and MT-1 (Fisher Scientific, Hampton, NH, USA, # NC1933275). SCC-17B was stained with MitoSox^TM^ Red Mitochondrial Superoxide Indicator according to the protocols described previously [18].

Slides were mounted using VECTASHIELD™ antifade mounting medium containing 4′,6-diamidino-2-phenylindole (DAPI) (Vector Laboratories, Burlingame, CA, USA, #H1200). Confocal images for immunofluorescence were acquired on a Zeiss LSM 700 microscope (Zeiss, Oberkochen, Germany) and analyzed in ImageJ [15]. A rolling-ball algorithm (radius, 50 pixels) was first applied for background correction, followed by Otsu automatic thresholding. Threshold parameters were held constant across all images within each experiment, consistent with prior methodology [25]. To account for differences in cell density, mean fluorescence intensity per field was normalized to the DAPI signal of the corresponding image. Quantification was carried out by an investigator blinded to treatment assignment.

### 2.6. Analysis of Apoptosis

Apoptotic cells were assessed using a terminal deoxynucleotidyl transferase-mediated dUTP nick end labeling (TUNEL) assay kit (Cell Signaling Technology, #25879) according to the manufacturer’s instructions.

### 2.7. Adenosine Triphosphate (ATP) Detection

NHOK and HOK-16B were seeded at approximately 1.5 × 10^5^ cells per well in 6-well plates containing 2 mL of medium. After overnight attachment, cultures received one of four treatments: PBS vehicle, GV1001 (10 μg/mL), 5-FU (200 μmol/L), or their combination, for 48 h. Intracellular ATP was then measured with a luminescence-based detection kit (Cayman Chemical, Ann Arbor, MI, USA, #700410) per the manufacturer’s instructions [18].

### 2.8. Lipid-GV1001 Binding Analysis

Ninety-six-well microplates (Genesee Scientific, El Cajon, CA, USA, #25-109) were coated with 2.5 µg per well of one of the following: cardiolipin (CL; C1649, MilliporeSigma), phosphatidylinositol 4,5-bisphosphate (PI(4,5)P_2_; Echelon Biosciences, #P-4508, Salt Lake City, UT, USA) or phosphatidylcholine (PC; MilliporeSigma, #P3556). PI(4,5)P_2_ and PC were used as negative control lipids. Plates were incubated overnight at 4 °C. Wells were then blocked for 2 h with 4-(2-hydroxyethyl)-1-piperazineethanesulfonic acid (HEPES) buffer (pH 7.4; H3375, MilliporeSigma) containing 2% (*w*/*v*) bovine serum albumin (BSA). Plates were washed three times with HEPES buffer containing 0.05% (*v*/*v*) Tween-20 (P1379, MilliporeSigma). GV1001 was added to the wells and incubated for 2 h, followed by three washes with HEPES-Tween buffer. An anti-GV1001 antibody (GemVax/Kael, Inc., Seongnam-si, Republic of Korea) was then added and incubated for 1 h, followed by three additional washes with HEPES-Tween buffer. Color development was achieved using 3,3′,5,5′-Tetramethylbenzidine (TMB, 860336, MilliporeSigma, Burlington, MA, USA) substrate, and the reaction was terminated with sulfuric acid. Absorbance was read at 490 nm excitation and 520 nm emission on a Bio-Rad PR4100 spectrophotometer (Bio-Rad, Hercules, CA, USA), as previously described [26]. Relative binding was calculated as a percentage (%) = (F − F_blank_)/(F_max_ − F_blank_) × 100, and the resulting values were indicated as percentage binding and were fitted to a sigmoidal dose–response curve.

### 2.9. GV1001 Protection-Inhibition Assay for Cell Proliferation and Mitochondrial ROS

NHOK and SCC-17B were seeded in 6-well plates and allowed to attach overnight (Day 0). On Day 1, fresh medium containing one of six treatment conditions was added: (i) vehicle control, (ii) GV1001 (10 µg/mL), (iii) 5-FU (125 µM), (iv) 5-FU (250 µM), (v) 5-FU (125 µM) plus GV1001 (10 µg/mL), or (vi) 5-FU (250 µM) plus GV1001 (10 µg/mL). Media was replenished every 48 h without altering seeding density. Viable cell numbers were determined on Days 0, 2, and 4 by trypan-blue exclusion using a hemocytometer and were expressed as ×10^4^ cells per well. Triplicate wells were analyzed per condition in five independent experiments. For mitochondrial ROS assessment in SCC-17B, cells were exposed to PBS vehicle, GV1001 (10 μg/mL), 5-FU (200 μmol/L), or 5-FU with GV1001 for 48 h. Cells were then marked with MitoSox™ Red mitochondrial superoxide indicator (Invitrogen, Carlsbad, CA, USA, #M36008).

### 2.10. Statistical Analyses

GraphPad Prism 9 (GraphPad Software) was used for all graphing and statistical testing. Survival curves were compared by the Kaplan–Meier method. Longitudinal body-weight data and all other multi-group comparisons were evaluated with the Kruskal–Wallis test, followed by Dunn’s correction for pairwise contrasts, unless stated otherwise. Results are reported as mean ± standard error (SE). Statistical significance was set at *p* < 0.05. Each experiment was independently replicated a minimum of five times. An a priori power calculation, assuming continuous symmetric data with α = 0.05, β = 0.2, and 80% power, indicated that 7–8 mice per group would be sufficient to detect biologically relevant treatment effects.

## 3. Results

### 3.1. GV1001 Attenuated 5-FU-Induced Mucosal Injury in the Esophagus, Intestine, and Tongue

**Figure 1:**
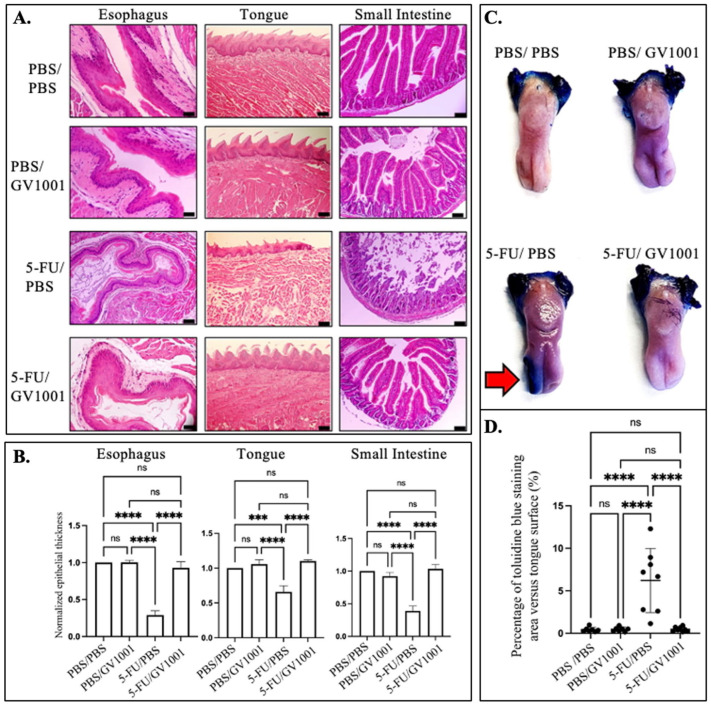
GV1001 attenuates 5-FU-induced mucosal injury in the upper and lower GI tract. (**A**) Representative H&E-stained sections of the esophagus, tongue, and small intestine. Scale bars: 50 μm. (**B**) Quantification of the epithelial thickness of the esophagus, tongue, and small intestine. Measurements were normalized to the mean epithelial thickness of the PBS/PBS control group. (**C**) Representative images of the tongues stained with toluidine blue. Dark blue regions on the anterior two-thirds of the tongue indicate ulcerated mucosa (red arrow). (**D**) Quantification of the ulcerated surface area. All data are presented as the mean ± SE (*n* = 8). Dots represent individual tongues. Significance levels are indicated: ns = not significant, *** *p* < 0.001, **** *p* < 0.0001.

Histological analysis of PBS/PBS- and PBS/GV1001-treated mice revealed intact mucosal architecture, including the stratified squamous epithelium of the tongue and esophagus, with orderly basal layers, well-defined tongue papillae, and continuous keratinization. Intestinal sections displayed slender villi and deep crypts (Figure 1A). In contrast, 5-FU/PBS-treated mice developed marked mucositis, characterized by focal tongue ulceration, epithelial thinning with loss of filiform papillae, esophageal epithelial atrophy with keratin desquamation, and significant villus blunting with crypt dropout in the small intestine. The 5-FU/GV1001 group demonstrated significant attenuation to these changes, with tissue morphology more closely resembling that of the control groups (Figure 1A,B and Figure S2).

Because mucosal ulceration is a hallmark of chemotherapy-induced oral mucositis, gross mucosal integrity was further assessed via toluidine blue (TB) staining of excised tongues. PBS/PBS- and PBS/GV1001-treated mice exhibited minimal TB retention, whereas 5-FU/PBS-treated mice showed extensive, contiguous TB uptake outlining broad ulcerated areas along the dorsal and lateral tongue surfaces. Notably, 5-FU/GV1001-treated mice showed significantly reduced TB retention at levels comparable to PBS/PBS-treated controls (Figure 1C,D and Figure S3).

5-FU treatment is associated with significant systemic toxicities, including body weight loss, leukopenia, and increased mortality in mice [27,28]. We therefore evaluated whether GV1001 could attenuate these adverse effects induced by 5-FU administration. As shown in Supplemental Figure S4, mice treated with 5-FU plus vehicle (5-FU/PBS) exhibited a significant decline in body weight beginning on Day 4 and persisting throughout the 14-day observation period compared with PBS/PBS-treated controls. While mice receiving 5-FU/GV1001 did not show a significant restoration of body weight relative to PBS/PBS controls, they demonstrated a significant survival benefit over the 14-day period, as four mice in the 5-FU/PBS group died before the endpoint of the experiment. Consistent with systemic toxicity, 5-FU/PBS-treated mice developed marked leukopenia. However, 5-FU/GV1001 mice showed significantly improved circulating leukocyte counts compared with the 5-FU/PBS treatment alone, although leukocyte levels did not fully recover to those observed in the PBS-treated control groups.

### 3.2. GV1001 Suppresses 5-FU-Induced NF-kB Activation and Pro-Inflammatory Cytokine Release

**Figure 2:**
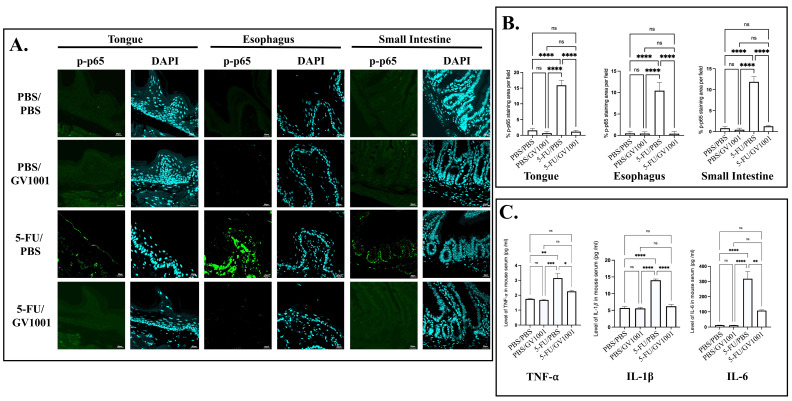
GV1001 suppresses NF-κB activation and reduces the production of pro-inflammatory cytokines. (**A**) Representative immunofluorescence staining images showing p-p65 (green) and DAPI (cyan) in the tongue, esophagus, and small intestine. Scale bar: 20 μm. (**B**) Quantification of p-p65 fluorescence intensity. (**C**) Serum levels of TNF-α, IL-1β, and IL-6 were measured by ELISA. All experiments were independently performed with at least five biological replicates. Statistical significance is indicated: ns = not significant, * *p* < 0.05, ** *p* < 0.01, *** *p* < 0.001, **** *p* < 0.0001.

Phosphorylated p65 (p-p65), a marker of transcriptionally active NF-κB, was markedly increased following 5-FU treatment. To determine whether GV1001 modulated this response, p-p65 expression was assessed via immunofluorescence. Mice treated with 5-FU/PBS exhibited a significant increase in p-p65 staining intensity in the tongue, esophagus, and small intestine, whereas treatment with 5-FU/GV1001 significantly mitigated this increase (Figure 2A,B). Consistent with the enhanced NF-κB signal, 5-FU/PBS-treated mice showed significantly elevated levels of the pro-inflammatory cytokines TNF-α, IL-1β, and IL-6 across all three tissues. In contrast, 5-FU/GV1001 treatment significantly suppressed these pro-inflammatory cytokines (Supplementary Figure S5). Serum ELISA results corroborated these findings, demonstrating significantly lower circulating levels of pro-inflammatory cytokines in 5-FU/GV1001-treated mice compared with the 5-FU/PBS group (Figure 2C).

### 3.3. GV1001 Mitigates 5-FU-Induced DNA Damage, Apoptosis, and Proliferation Arrest Across GI Mucosa

**Figure 3:**
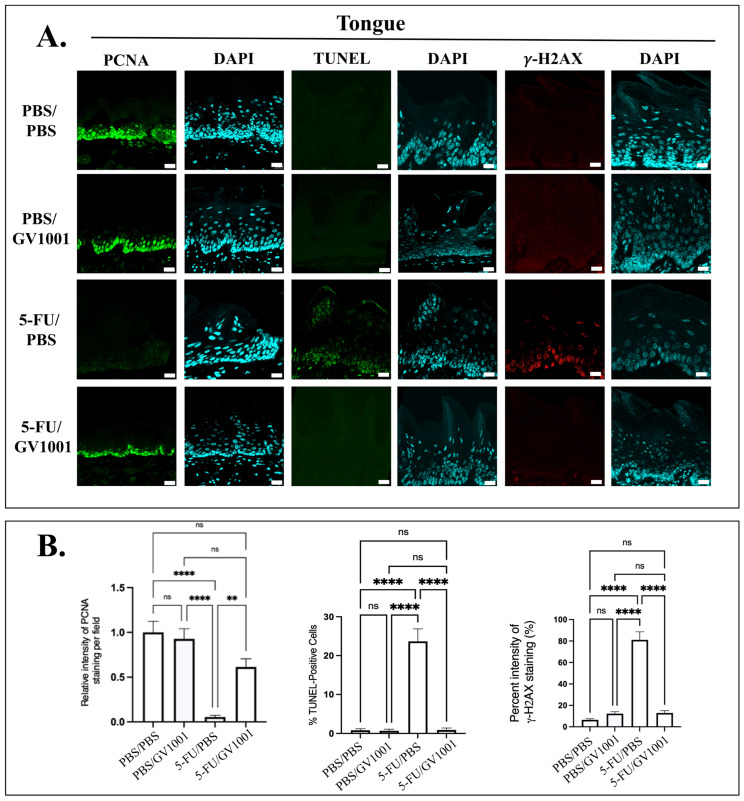
GV1001 mitigates 5-FU-driven proliferation arrest, DNA damage, and apoptosis in the oral mucosa. (**A**) Representative immunofluorescence images of tongue tissue. PCNA (green) marks proliferating cells, TUNEL staining (green) identifies apoptotic cells, γ-H2AX (red) indicates DNA double-strand breaks. Nuclei are counterstained with DAPI (cyan). Scale bar: 20 μm. (**B**) Quantification of PCNA, TUNEL, and γ-H2AX staining intensity in tongue tissue. All experiments were independently performed with at least five biological replicates. Statistical significance was indicated: ns = not significant, ** *p* < 0.01, **** *p* < 0.0001.

As shown in Figure 3 and Figure S6, tissues from 5-FU/PBS-treated mice exhibited increases in γ-H2AX-positive nuclei across the tongue, esophagus, and small intestine, indicating significant DNA double-strand breaks. This response was accompanied by increased TUNEL-positive staining in the tongue and esophageal epithelium, as well as the intestinal crypts, consistent with enhanced apoptosis. In parallel, proliferative capacity was significantly reduced, as evidenced by a decrease in the number of PCNA-positive basal cells. In contrast, 5-FU/GV1001-treated mice attenuated these genotoxic and apoptotic responses, with γ-H2AX and TUNEL positivity reduced to levels comparable to PBS/PBS-treated controls, alongside the restoration of PCNA-positive cell populations. Consistent with these in vivo findings, as shown in Supplementary Figure S7, in vitro analyses demonstrated that NHOK and HOK-16B exposed to 5-FU exhibited extensive γ-H2AX foci, whereas GV1001 co-treatment significantly reduced DNA damage to near-control levels.

### 3.4. GV1001 Attenuates 5-FU-Induced EMT In Vivo and In Vitro

**Figure 4:**
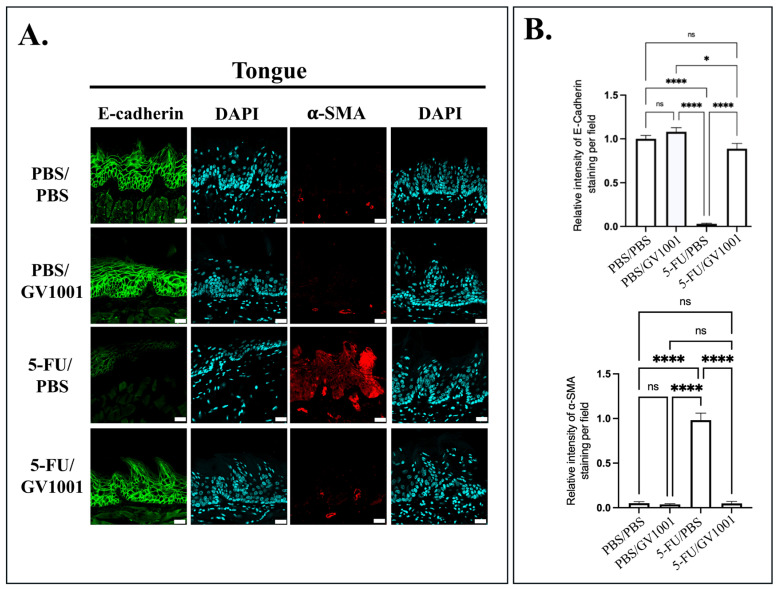
GV1001 preserves epithelial identity and suppresses mesenchymal transition in oral mucosa with 5-FU exposure. (**A**) Representative immunofluorescence images of tongue tissue. E-cadherin (green) marks epithelial adherence junctions, and α-SMA (red) identifies mesenchymal-like cells. Nuclei are counterstained with DAPI (cyan). Scale bar: 20 μm. (**B**) Quantification of E-cadherin and α-SMA fluorescence intensity in tongue tissue using ImageJ. All experiments were independently performed with at least five biological replicates. Statistical significance is indicated: ns = not significant, * *p* < 0.05, **** *p* < 0.0001.

5-FU is known to induce EMT in mucosal epithelium, characterized by the loss of E-cadherin and upregulation of mesenchymal markers, such as vimentin, N-cadherin, and α-SMA [13,29]. In vivo, the GI tract tissues from 5-FU/PBS-treated mice showed a marked reduction in E-cadherin expression and an increase in α-SMA in all analyzed sites. In contrast, GV1001 cotreatment attenuated these EMT-associated changes, restoring E-cadherin expression and suppressing mesenchymal markers to levels comparable to control (Figure 4 and Figure S8).

In the esophageal epithelium, 5-FU treatment also increased N-cadherin expression and significantly reduced the basal cell marker p63. Both alterations were reversed in 5-FU/GV1001-treated mice, which restored basal-layer p63 expression and reduced N-cadherin levels (Supplemental Figure S9). The protective effects extended to local inflammatory responses, where immunofluorescence for monocyte/macrophage antigen-2 (MOMA-2) revealed dense peri-epithelial monocyte/macrophage infiltration in the tongues of 5-FU/PBS-treated mice, whereas 5-FU/GV1001-treated tongues showed significantly reduced immune cell infiltration (Supplementary Figure S10). Consistent with these in vivo findings, in vitro studies demonstrated that NHOK and HOK-16B exposed to 5-FU for 48 h exhibited increased α-SMA and vimentin expression with concomitant loss of E-cadherin. Co-treatment with GV1001 significantly reversed these EMT-associated changes, restoring epithelial marker expression and suppressing mesenchymal markers. (Supplementary Figure S11).

### 3.5. GV1001 Mitigates the Cytotoxic Effect of 5-FU and Preserves Mitochondrial Integrity and Bioenergetic Function of Normal Human Keratinocytes

**Figure 5:**
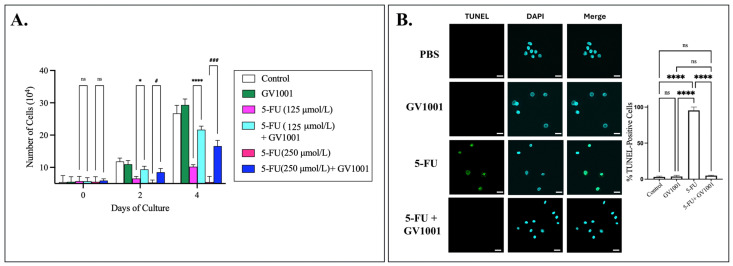
GV1001 mitigates the cytotoxic effects of 5-FU in normal human oral keratinocytes. (**A**) NHOK were treated with PBS (control), GV1001 alone (10 µg/mL), 5-FU at 125 µmol/L or 250 µmol/L, or the corresponding 5-FU + GV1001 combinations. Cell numbers were quantified on Days 0, 2, and 4 using a hemocytometer and are plotted as ×10^4^ cells per well (mean ± SE). Compared with the 5-FU (125 μmol/L) group: * *p* < 0.05, **** *p* < 0.0001; compared with the 5-FU (250 μmol/L) group: # *p* < 0.05 and ### *p* < 0.001. (**B**) Representative immunofluorescence images of TUNEL staining (green) and nuclei counterstained with DAPI (cyan) in NHOK. Quantification of TUNEL fluorescence intensity was performed using ImageJ. Statistical significance is indicated: ns = not significant, **** *p* < 0.0001. Scale bar: 20 μm. Data represents at least five independent experiments.

As shown in Figure 5, 5-FU significantly suppressed NHOK proliferation and increased cell death, whereas GV1001 markedly attenuated these effects, indicating that GV1001 protects normal keratinocytes from the cytotoxic effects of 5-FU.

**Figure 6:**
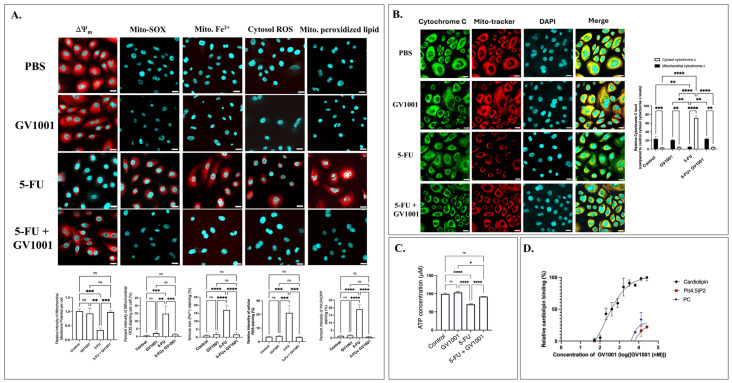
GV1001 preserves mitochondrial integrity and function in 5-FU-treated NHOK. (**A**) Representative immunofluorescence images of NHOK showing mitochondrial membrane potential (ΔΨ_m_, red), Mito-SOX (mitochondrial ROS, red), Mito-FerroGreen (Mito. Fe^2+^, red), cytosolic ROS (red), and mitochondrial peroxidized lipid (MitoPeDPP, red). Nuclei were counterstained with DAPI (cyan). (**B**) Representative immunofluorescence images showing cytochrome c (green), MitoTracker Red dye (red), and DAPI (cyan). Data are mean ± SE. Scale bar: 20 μm. (**C**) Intracellular ATP in NHOK. Significance levels are indicated: ns = not significant, * *p* < 0.05, ** *p* < 0.01, *** *p* < 0.001, **** *p* < 0.0001. (**D**) ELISA-based dose–response curves showing GV1001 binding to CL, PI(4,5)P2, or PC. Data were fitted using a four-parameter logistic model and are presented as mean ± SE. Quantification was performed using ImageJ. Statistical Data represents at least five independent experiments.

As 5-FU is known to exhibit cytotoxicity partly via mitochondrial damages, we analyzed the effect of 5-FU and GV1001, alone or in combination, on the mitochondrial integrity in NHOK and nontumorigenic immortalized HOK-16 cells. Immunofluorescence analysis of both NHOK and HOK-16B revealed that 5-FU profoundly disrupted mitochondrial homeostasis. As shown in Figure 6A,B, in NHOK, 5-FU markedly reduced mitochondrial membrane potential (ΔΨ_m_) and MitoTracker Red retention in NHOK, while significantly increasing mitochondrial superoxide (MitoSOX), cytoplasmic ROS, mitochondrial Fe^2+^ accumulation, and lipid peroxidation. Co-treatment with GV1001 normalized ΔΨ_m_, restored MitoTracker retention, and significantly reduced mitochondrial oxidative stress. While 5-FU treatment alone resulted in the release of cytochrome into the cytosol, this was notably prevented by GV1001, which maintained cytochrome c colocalization with MitoTracker, indicating intact mitochondrial sequestration. Similar protective effects were observed in HOK-16B (Supplemental Figure S12).

Consistent with these structural and redox effects, 5-FU alone significantly reduced intracellular ATP levels, while concurrent treatment with GV1001 restored ATP production to near control levels (Figure 6C). To further assess mitochondrial respiratory capacity, immunofluorescence staining of oxidative phosphorylation complexes I–IV was performed (Supplemental Figure S13). 5-FU caused a significant reduction of complex I (NDUFS1), complex II (SDHB), complex III (UQCRFS1), and complex IV (COX IV) compared to the control, whereas GV1001 mitigated this 5-FU-induced reduction for both NHOK and HOK-16B.

Cardiolipin (CL), a phospholipid predominantly localized in the inner mitochondrial membrane (IMM), is essential for maintaining membrane integrity and supporting efficient electron transport and ATP production. The synthetic tetrapeptide and elamipretide (SS-31) have been shown to enhance mitochondrial function through direct binding to cardiolipin [30]. Consistently, our prior work demonstrated that GV1001 interacts with cardiolipin in human renal epithelial cells, preserving electron transport chain (ETC) function [18]. To determine whether GV1001 directly interacts with cardiolipin in the mitochondria of human oral epithelial cells, we quantified GV1001-CL binding using an ELISA-based approach. GV1001 bound to CL in a concentration-dependent manner, with an EC_50_ of 179.2 nmol/L (Figure 6D), while the control lipids PI(4,5)P2 and PC showed minimal binding, with interactions observed at high GV1001 concentrations. Together, these findings demonstrate that GV1001 localizes to the mitochondria and binds CL with nanomolar affinity, supporting a direct lipid-peptide interaction as a mechanism underlying its mitochondrial protective effects.

### 3.6. GV1001 Mitigated 5-FU-Induced Suppression of Cell Proliferation in NHOK but Did Not Reverse the Growth Inhibition in SCC-17B Squamous Cell Carcinoma Cells

**Figure 7:**
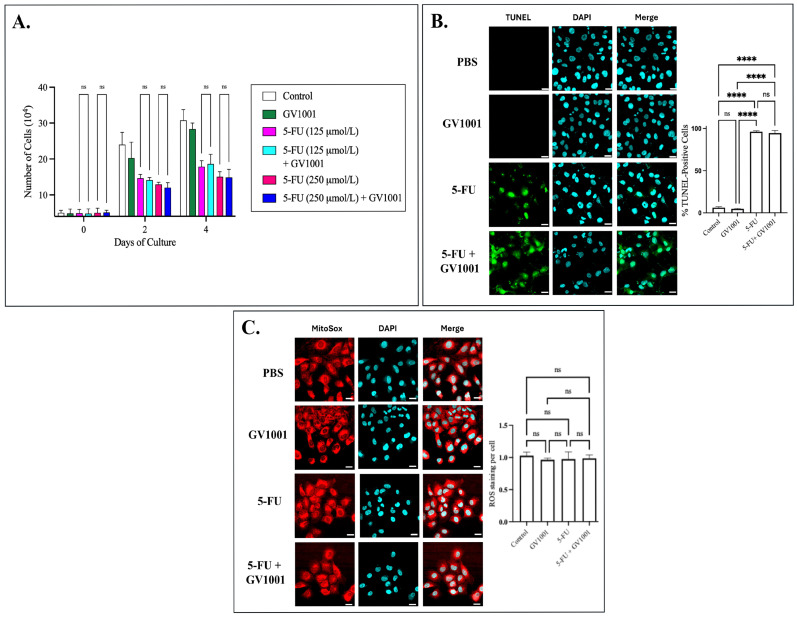
GV1001 does not protect human squamous carcinoma cells during 5-FU exposure. (**A**) SCC-17B were treated with PBS (control), GV1001 alone (10 µg/mL), 5-FU at 125 µmol/L or 250 µmol/L, or the corresponding 5-FU + GV1001 combinations. Cell numbers were quantified on Days 0, 2, and 4 using a hemocytometer and are plotted as ×10^4^ cells per well (mean ± SE). (**B**) Representative immunofluorescence images of TUNEL staining (green) and nuclei counterstained with DAPI (cyan) in SCC-17B. Quantification of TUNEL fluorescence intensity was performed using ImageJ. (**C**) Representative immunofluorescence images of SCC-17B stained for MitoSox (mtROS) levels (red) and nuclei counterstained with DAPI (cyan). Quantification of MitoSox fluorescence intensity was performed with ImageJ. Statistical significance is indicated: ns = not significant, **** *p* < 0.0001. Scale bar: 20 μm. Data represents at least five independent experiments.

An essential translational question is whether GV1001 preserves host tissues without diminishing the anti-tumor efficacy of 5-fluorouracil (5-FU). To address this, we examined the effects of 5-FU, alone or in combination with GV1001, on the proliferative capacity and mitochondrial integrity of SCC-17B. As shown in Figure 7A, GV1001 did not reverse the 5-FU-induced proliferation arrest in SCC-17B cells. TUNEL staining further confirmed that 5-FU induced apoptosis in SCC-17B, and co-treatment with GV1001 did not attenuate this effect (Figure 7B). Additionally, no significant differences in mitochondrial ROS (mtROS) levels were observed among the treatment groups in SCC-17B cells, including those treated with 5-FU in the presence or absence of GV1001 (Figure 7C).

## 4. Discussion

CIM represents a complex convergence of oxidative injury, genotoxic stress, and inflammatory amplification that collectively destabilizes epithelial homeostasis throughout the GI tract [1,31]. While 5-FU remains a cornerstone of anti-neoplastic therapy, effective strategies to prevent and mitigate associated CIM remain limited, with current interventions remaining largely supportive [1]. In this study, utilizing a murine model of 5-FU-induced CIM, we demonstrated that GV1001 robustly attenuates mucositis by preserving mitochondrial integrity, thereby restraining oxidative and inflammatory signaling, along with maintaining epithelial regenerative capacity and architecture.

Gross histological examination revealed that GV1001 significantly diminished 5-FU-induced epithelial disruption across the GI tract, including the tongue, esophagus, and small intestine. These structural improvements were accompanied by enhanced survival and improved white blood cell counts, supporting a systemic protective effect. Although GV1001 did not fully prevent chemotherapy-associated weight loss, it still provided slight protection against weight loss. The attenuation of these lesions by GV1001 can be attributed partially to the downregulation of the NF-κB signaling pathway [18,32]. GV1001 reduces cytosolic ROS and mitogen-activated protein kinase (MAPK) signaling, both of which typically contribute to the activation of NF-κB [33]. Further, serum cytokine profiling showed marked suppression of pro-inflammatory cytokines IL-1β, IL-6, and TNF-α, reinforcing GV1001’s recognized inhibition of the p38 MAPK and NF-κB pathways [34]. In addition to inflammatory signaling, 5-FU is known to trigger epithelial cell death, suppress proliferation, and induce excessive double-strand DNA breaks [11,35]. GV1001 prevented epithelial cell death, restored proliferation, and reduced double-stranded DNA breaks, which are consistent with previous reports that GV1001 can mitigate DNA damage in irradiated keratinocytes and mitigate oxidative stress in hepatocytes and neural stem cells. Our findings parallel work in a G-protein-coupled estrogen receptor model of intestinal injury, which supports the premise that limiting crypt DNA damage significantly blunts mucositis severity [31]. Furthermore, 5-FU induces an EMT phenotype in mucosal epithelial cells [33]. GV1001’s ability to prevent EMT phenotype with 5-FU treatment mirrors reports that it suppresses transforming growth factor beta (TGF-β)-driven EMT across prostate, dermal, and keratinocyte systems [36]. Furthermore, lymphopenia in patients is associated with poorer cancer survival outcomes [37], and the inhibition of 5-FU-induced WBC depletion observed with GV1001 treatment may contribute to improved prognosis. Collectively, these anti-inflammatory, anti-EMT and hematopoietic-protective effects distinguish GV1001 from growth factor agonists such as palifermin, which primarily stimulate epithelial proliferation and re-epithelialization, without addressing the inflammatory and mesenchymal drivers of mucosal pathology [38].

To elucidate the key mechanisms underlying GV1001-mediated protection against CIM, we examined its impact on 5-FU-mediated mitochondrial dysfunction, as growing evidence indicates that early mitochondrial injury acts as a critical upstream integrator and propagator of these inflammatory pathways [39]. In mucositis pathogenesis, mitochondrial impairment plays a pivotal role in linking genotoxic stress to ROS generation, inflammatory signaling, and epithelial barrier disruption [40,41]. Notably, 5-FU exerts a dual pressure on the respiratory chain by disrupting the synthesis of nuclear-encoded ETC subunits and upregulating the expression of fission proteins, such as dynamin-related protein 1 (Drp1), while downregulating fusion proteins important for the repair of damaged mitochondria [42]. These effects contribute to the depletion of respiratory complexes and the dissipation of mitochondrial potential seen in NHOK and HOK-16B. Together, the disruption of ETC proteins and fission-coupled mitophagy converge to deplete ETC complexes and ATP production. Furthermore, oxidative injury to the inner mitochondrial membrane phospholipid cardiolipin (CL) destabilizes critical CL-ETC interactions. The peroxidation of CL disrupts respiratory chain super-complex assembly, thereby impairing ETC function [43]. Once oxidized, CL loses its ability to anchor cytochrome c to the inner mitochondrial membrane, facilitating its release into the cytosol and the subsequent activation of pro-apoptotic factors [44]. In parallel, ETC collapse and CL oxidation reduce ATP synthesis, while sustained mtROS production promotes the opening of the mitochondrial permeability transition pore (mPTP), further dissipating the ΔΨm and amplifying cytosolic ROS signaling [45]. Elevated cytosolic ROS activates redox-sensitive transcription factors such as NF-κB. Persistent NF-κB signaling, along with mtROS, promotes the EMT phenotype, or when oxidative stress exceeds cellular antioxidant capacity, engages apoptotic pathways [46]. Based on this context, our data demonstrate that GV1001 robustly safeguards mitochondrial integrity upstream of this injury cascade by protecting CL from oxidation. Our ELISA data showed a strong, preferential GV1001-CL-specific interaction relative to other membrane lipids, supporting the peptide’s ability to selectively protect CL [18]. This protection likely causes GV1001 to shield CL from peroxidation, thus normalizing ΔΨm, restoring ATP production to near-baseline levels, and preventing cytochrome c release into the cytosol in both NHOK and HOK-16B, thus preventing cell death. Functionally, mitochondrial stabilization by GV1001 resulted in the suppression of NF-κB nuclear translocation, attenuation of apoptosis, and the coordinated preservation of the crypt-villus architecture and epithelial continuity. Importantly, these downstream protective effects were observed consistently across both in vivo and in vitro models, supporting a unified mitochondria-centric mechanism underlying GV1001-mediated epithelial protection.

GV1001 is a synthetic hTERT-derived peptide, and there is currently no evidence that this fragment is generated endogenously or accumulates at functionally relevant levels under physiological conditions. It was reported that mitochondrial TERT can protect mitochondrial DNA under specific stress conditions [47]. As GV1001 demonstrated 5-FU-induced DNA damages and its direct binding to mitochondrial phospholipid, cardiolipin, GV1001 would protect against DNA damages like that of full-length mitochondrial TERT.

A translational concern for host-directed cytoprotective strategies in the setting of chemotherapy is whether protection of normal tissues comes at the expense of anti-tumor efficacy. In vitro, we demonstrated that GV1001 selectively preserved the proliferative capacity and viability of NHOK or noncancerous immortalized HOK-16B exposed to 5-FU, while having minimal impact on the chemo-toxic effects of 5-FU in oral cancer cells, SCC-17B. This differential response indicates that GV1001 does not broadly antagonize the cytotoxic activity of 5-FU but rather confers context-dependent protection restricted to non-transformed epithelial cells. Such selectivity is essential for the clinical viability of any adjunctive therapy aimed at mitigating CIM. Mechanistically, this differential response likely reflects fundamental differences in mitochondrial dependence, redox homeostasis, and stress-response wiring between normal and cancerous epithelial cells. Normal oral keratinocytes rely on intact mitochondrial signaling and redox balance to sustain proliferation and tissue renewal, rendering them particularly vulnerable to 5-FU-induced mitochondrial dysfunction and ROS-driven apoptosis [12]. In contrast, SCC-17B, like many carcinomas, exhibits altered mitochondrial metabolism, elevated baseline oxidative stress tolerance and generation, along with dysregulated apoptotic checkpoints, which may limit the capacity of mitochondrial stabilization alone to override 5-FU-mediated growth inhibition [48,49]. Our data shows that mtROS levels remain high even in the presence of GV1001, supporting the premise that GV1001 does not protect these cancer cells, likely due to their elevated baseline oxidative burden compared to normal cells.

While our study is the first to demonstrate GV1001’s ability to blunt CIM by preserving mitochondrial function, suppressing inflammasome activation, and restraining EMT, several limitations should be acknowledged. Mucositis resulting from epithelial damage represents a significant clinical challenge in cancer chemotherapy, not only with 5-fluorouracil (5-FU) but also with other agents, including doxorubicin, liposomal doxorubicin (Doxil), tyrosine kinase inhibitors (TKIs) such as cabozantinib, irinotecan (SN-38), and methotrexate. Therefore, the protective effects of GV1001 against a broader range of chemotherapeutic agents warrant comprehensive investigation in future studies. In addition, nutritional supplements such as Healios (developed by Enlivity) are currently used to alleviate oral mucositis and esophagitis in cancer patients in clinical settings [50]. The potential synergistic effects and underlying mechanisms of GV1001 in combination with such agents in mitigating GI mucositis merit further exploration.

The present work primarily focused on acute injury endpoints in high-dose models, which, although well-validated for mucositis induction, may not fully recapitulate the dosing kinetics encountered in clinical oncology. Moreover, although our data suggest that GV1001 protects CL from oxidative damage, the precise molecular interaction between the peptide and mitochondrial lipids requires further investigation. Future studies should employ complementary biophysical and biochemical approaches to clarify how GV1001 stabilizes CL and respiratory complexes. Finally, while our in vitro observations indicate that GV1001 does not protect squamous carcinoma cells during 5-FU co-treatment, these findings must be validated in vivo to confirm the maintenance of anti-tumor efficacy. With these limitations in mind, the results highlight GV1001 as a promising candidate for mitigating the mitochondrial and inflammatory drivers of chemotherapy-induced mucositis.

## 5. Conclusions

GV1001 significantly attenuated 5-FU-induced chemotherapy-associated mucositis by reducing epithelial injury, inflammation, DNA damage, apoptosis, and epithelial–mesenchymal transition across the gastrointestinal tract, while improving leukocyte counts and survival. Mechanistically, GV1001 preserved mitochondrial integrity by limiting mitochondrial oxidative stress, restoring ATP production, and stabilizing mitochondrial membranes through preferential interaction with CL. Importantly, GV1001 selectively protected normal epithelial cells without reducing the cytotoxic effects of 5-FU in squamous carcinoma cells, supporting its potential as a promising adjunctive strategy to mitigate chemotherapy-induced mucosal injury.

## Data Availability

Data is available upon request to the corresponding author.

## Supplementary Materials

The following supporting information can be downloaded at: https://www.mdpi.com/article/10.3390/cells15090774/s1. Figure S1: Experimental design and treatment schedule for the 5-FU–induced mucositis mouse model; Figure S2. GV1001 alleviates 5-FU-mediated mucosal damage in the upper and lower GI tract; Figure S3. Toluidine blue staining showcasing GV1001-mediated protection against 5-FU-induced ulceration of the oral mucosa; Figure S4. GV1001 improves survival and attenuates leukopenia in 5-FU-treated mice with modest effects on body weight; Figure S5. GV1001 dampens 5-FU–induced inflammatory cytokines in oral and gastrointestinal mucosa; Figure S6. GV1001 mitigates 5-FU–induced apoptosis and DNA damage while preserving proliferative capacity in esophageal and intestinal epithelium; Figure S7. GV1001 reduces 5-FU–induced DNA double-strand-break formation in oral epithelial cell lines; Figure S8. GV1001 preserves epithelial identity and suppresses mesenchymal transition in oral mucosa with 5-FU exposure; Figure S9. GV1001 preserves the basal progenitor marker p63 and prevents N-cadherin induction in 5-FU–treated esophageal epithelium; Figure S10. GV1001 limits 5-FU–induced infiltration of monocytes/macrophages in the tongue; Figure S11. GV1001 counteracts 5-FU–driven epithelial-to-mesenchymal transition (EMT) in normal keratinocytes (NHOK) and HOK-16B cells; Figure S12. GV1001 preserves mitochondrial integrity and function in 5-FU-treated oral keratinocytes HOK-16B; Figure S13. GV1001 preserves mitochondrial respiratory-chain integrity after 5-FU exposure in oral epithelial cell lines.

## Abbreviations

The following abbreviations are used in this manuscript:

5-FU	5-Fluorouracil
α-SMA	Alpha Smooth Muscle Actin
ATP	Adenosine Triphosphate
BSA	Bovine Serum Albumin
CL	Cardiolipin
CIM	Chemotherapy-induced Mucositis
COX IV	Cytochrome c Oxidase Subunit IV
DAPI	4′,6-Diamidino-2-Phenylindole
DMEM	Dulbecco’s Modified Eagle Medium
DNA	Deoxyribonucleic Acid
EC50	Half-Maximal Effective Concentration
ELISA	Enzyme-Linked Immunosorbent Assay
EMT	Epithelial–Mesenchymal Transition
ETC	Electron Transport Chain
FBS	Fetal Bovine Serum
GI	Gastrointestinal
H&E	Hematoxylin and Eosin
HEPES	4-(2-Hydroxyethyl)-1-Piperazineethanesulfonic Acid
HOK-16B	Human Oral Keratinocyte-16B
hTERT	Human Telomerase Reverse Transcriptase
IF	Immunofluorescence
IL-1β	Interleukin-1 Beta
IL-6	Interleukin-6
i.p.	Intraperitoneal
i.v.	Intravenous
MAPK	Mitogen-Activated Protein Kinase
MOMA-2	Monocyte/Macrophage Antigen-2
mtROS	Mitochondrial Reactive Oxygen Species
NF-κB	Nuclear Factor Kappa-B
NHOK	Normal Human Oral Keratinocytes
NDUFS1	NADH Dehydrogenase Fe-S Protein 1
PBS	Phosphate-Buffered Saline
PC	Phosphatidylcholine
PCNA	Proliferating Cell Nuclear Antigen
PFA	Paraformaldehyde
PI(4,5)P_2_	Phosphatidylinositol-4,5-bisphosphate
ROS	Reactive Oxygen Species
SCC-17B	Squamous Cell Carcinoma Cell Line-17B
SDHB	Succinate Dehydrogenase Complex Iron Sulfur B
TB	Toluidine Blue
TGF-β	Transforming Growth Factor Beta
TMB	3,3′,5,5′-Tetramethylbenzidine
TNF-α	Tumor Necrosis Factor Alpha
TUNEL	Terminal Deoxynucleotide Transferase-Mediated dUTP Nick End Labeling
UQCRFS1	Ubiquinol-Cytochrome c Reductase, Rieske Iron-Sulfur Polypeptide 1
WBC	White Blood Cells
SE	Standard error
