# Supplementary material for: GV1001, hTERT Peptide Fragment, Prevents 5-Fluorouracil-Induced Mucositis by Inhibiting Mitochondrial Damages
Source: Cells. 2026 Apr 25;15(9):774. doi: 10.3390/cells15090774 (PMC13162839; doi:10.3390/cells15090774)
Supplement: Supplementary file 1 [file cells-15-00774-s001.zip › cells-4233263-supplementary.pdf]

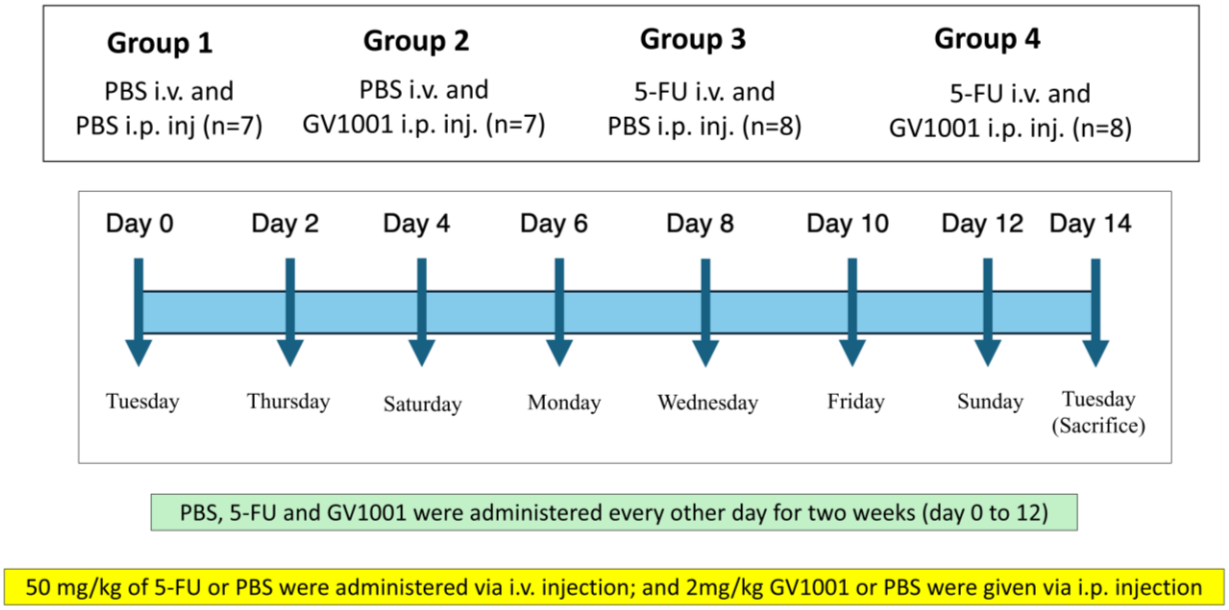

Figure S1. Experimental design and treatment schedule for the 5-FU–induced mucositis mouse model. Four treatment groups were established: Group 1 received PBS intravenously (i.v.) and PBS intraperitoneally (i.p.) (n = 7); Group 2 received PBS i.v. and GV1001 i.p. (n = 7); Group 3 received 5-fluorouracil (5-FU) i.v. and PBS i.p. (n = 8); and Group 4 received 5-FU i.v. and GV1001 i.p. (n = 8). Treatments were administered every other day from Day 0 to Day 12, as depicted in the timeline (Days 0, 2, 4, 6, 8, 10, and 12). Animals were euthanized on Day 14.

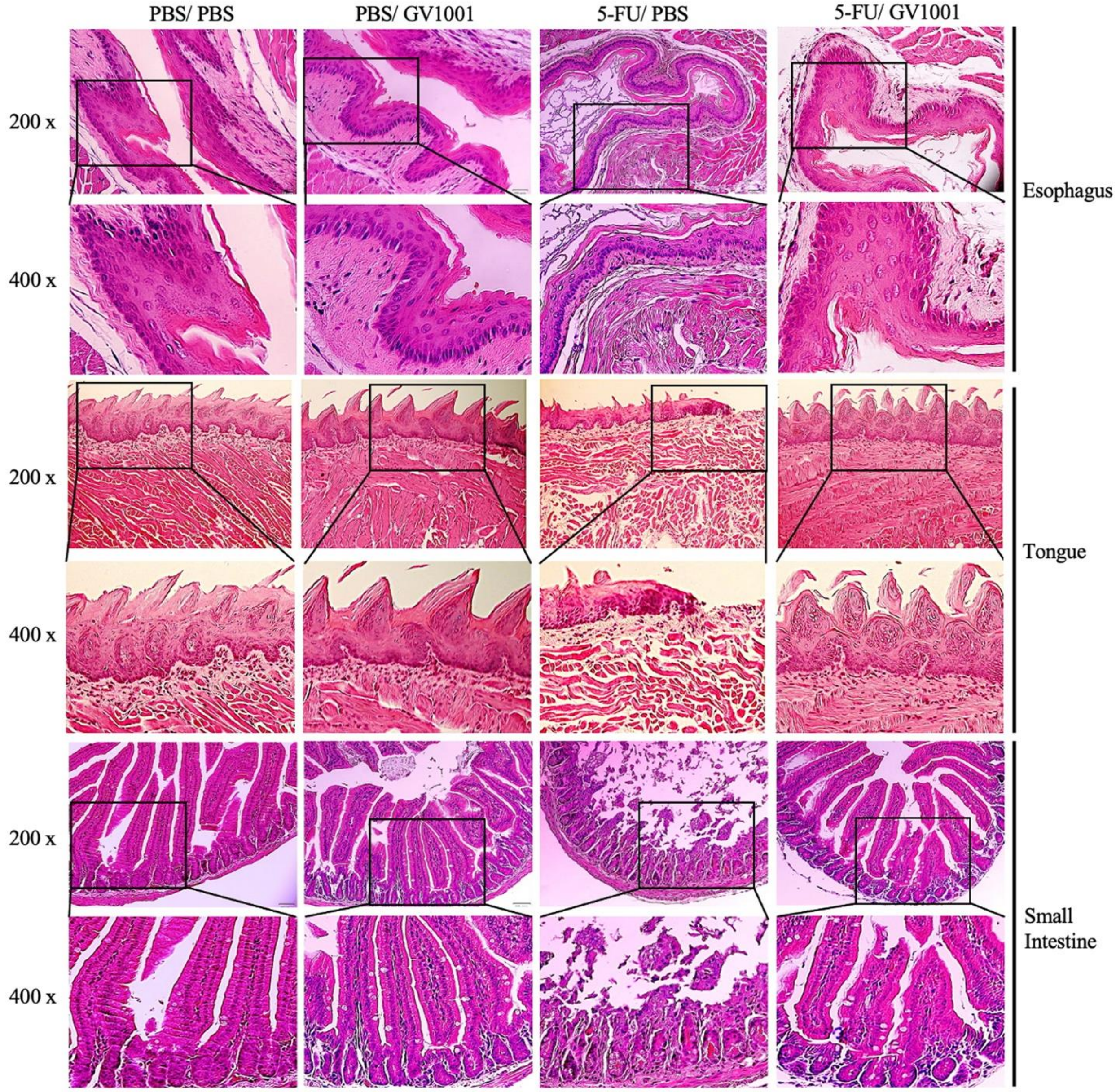

Figure S2. GV1001 alleviates 5-FU-mediated mucosal damage in the upper and lower GI tract. Representative H&E-stained sections of the esophagus, tongue, and small intestine illustrating histopathological features with lower and higher magnification (200x and 400x) in mice exposed to PBS/ PBS, PBS/ GV1001, 5-FU/ PBS, or 5-FU/ GV1001.

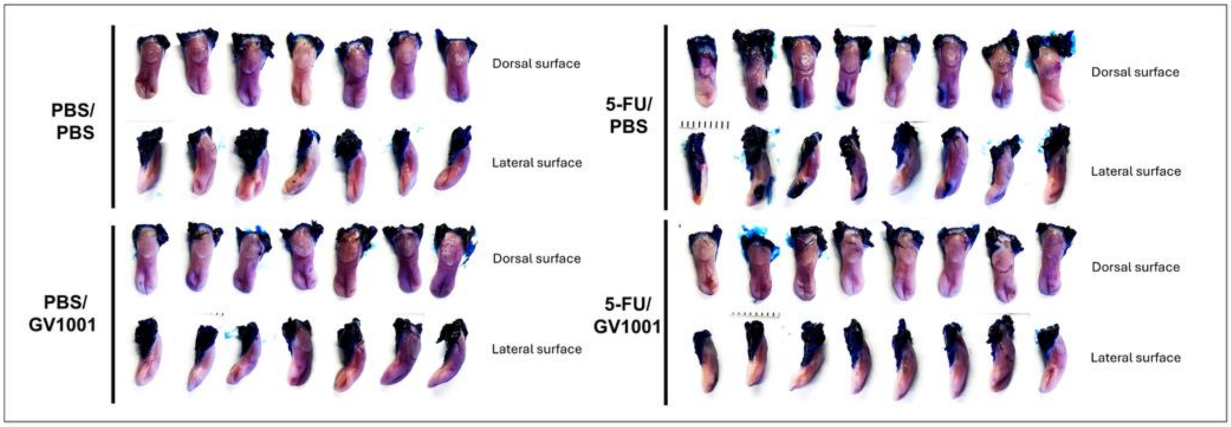

Figure S3. Toluidine blue staining showcasing GV1001-mediated protection against 5-FU-induced ulceration of the oral mucosa. Representative dorsal and lateral tongue surfaces collected on Day 14 from mice treated with PBS/PBS, PBS/GV1001, 5-FU/PBS, or 5-FU/GV1001. Toluidine blue staining demarcates areas of mucosal ulceration, with dark blue regions indicating epithelial barrier disruption on the anterolateral surface of the tongue.

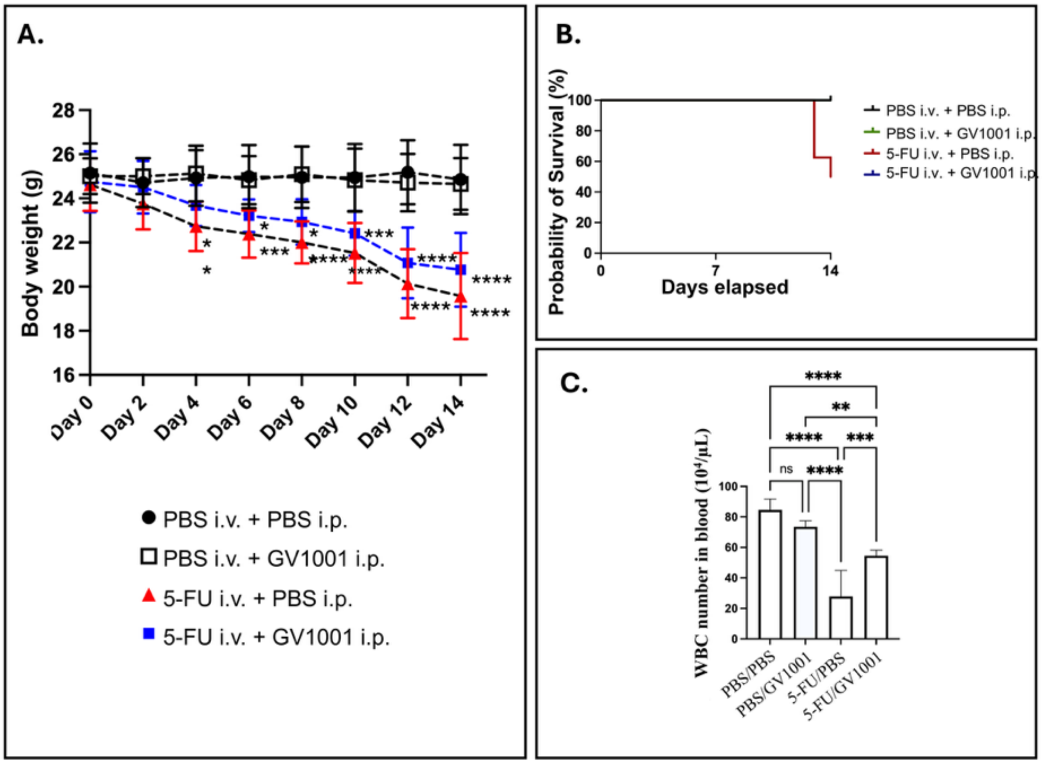

Figure S4. GV1001 improves survival and attenuates leukopenia in 5-FU-treated mice with modest effects on body weight. (**A**) Longitudinal body weight measurement of mice treated PBS intravenously (i.v.) plus PBS intraperitoneally (i.p.) (PBS/PBS), PBS i.v. plus GV1001 i.p. (PBS/GV1001), 5-fluorouracil (5-FU) i.v. plus PBS i.p. (5-FU/PBS), or 5-FU i.v. plus GV1001 i.p. (5-FU/GV1001). Body weights were recorded every other day for 14 days. Statistical significance is indicated versus PBS/PBS at the indicated time point. (**B**) Kaplan-Meier survival analysis over the duration of the experiment. (**C**) Peripheral white blood cell (WBC) counts were measured on the day of sacrifice. Significance indicated: ns = not significant, * $p< 0.05$, ** $p< 0.01$, *** $p< 0.001$, **** $p< 0.0001$.

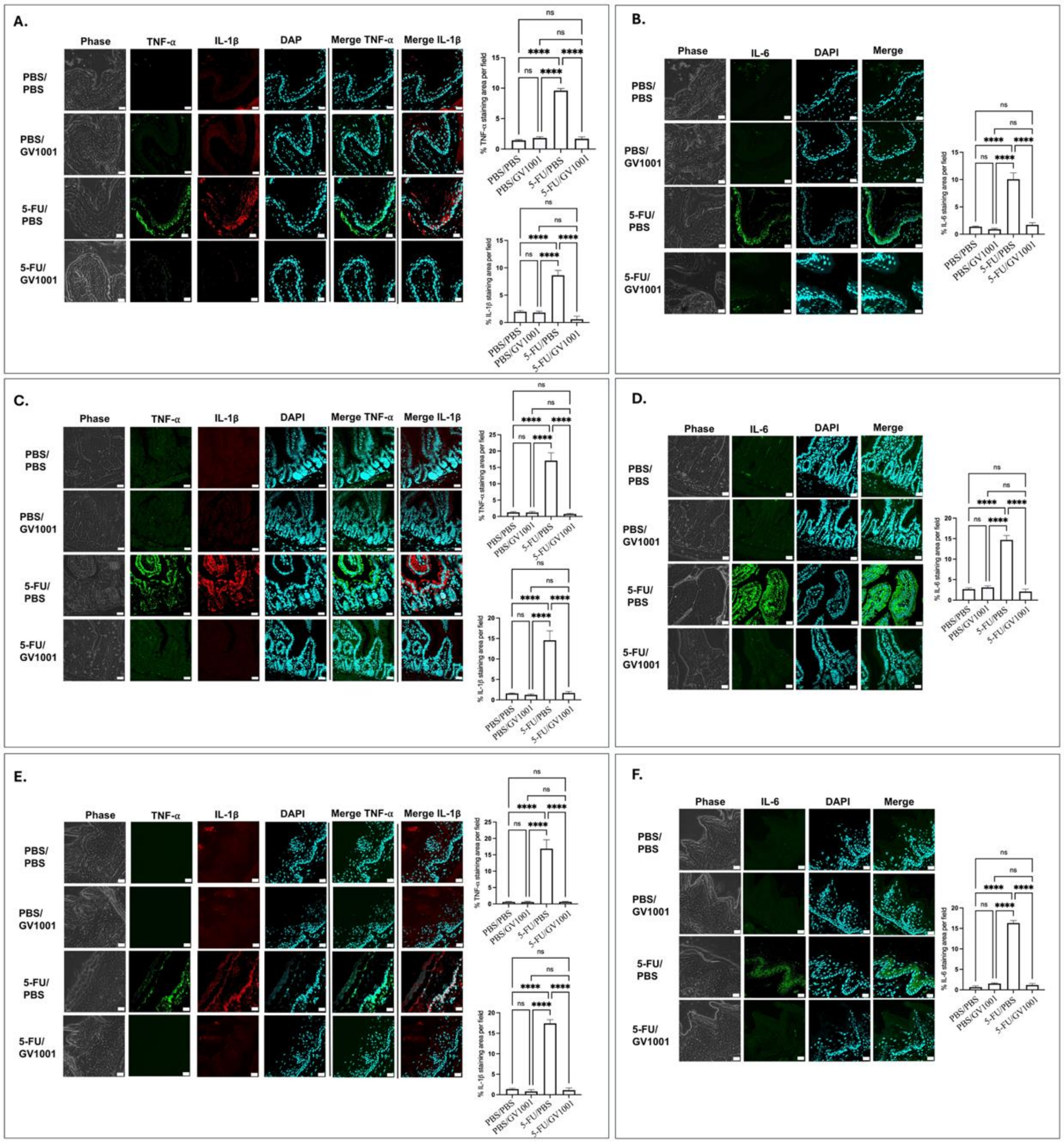

Figure S5. GV1001 dampens 5-FU–induced inflammatory cytokines in oral and gastrointestinal mucosa. (**A** and **B**) Representative immunofluorescence staining of esophageal mucosa showing TNF-α/IL-1β (**A**) and IL-6 (**B**). (**C** and **D**) Representative staining of tongue epithelium for TNF-α/IL-1β (**C**) and IL-6 (**D**). (**E** and **F**) Representative staining of small-intestinal villi for TNF-α/IL-1β (**E**) and IL-6 (**F**). Cytokines are visualized in green (TNF-α or IL-6) and red (IL-1β); nuclei are counter-stained with DAPI (cyan). Scale bar: 20 µm. Significance levels: ns = not significant, *p < 0.05, **p < 0.01, ***p < 0.001, ****p < 0.0001.

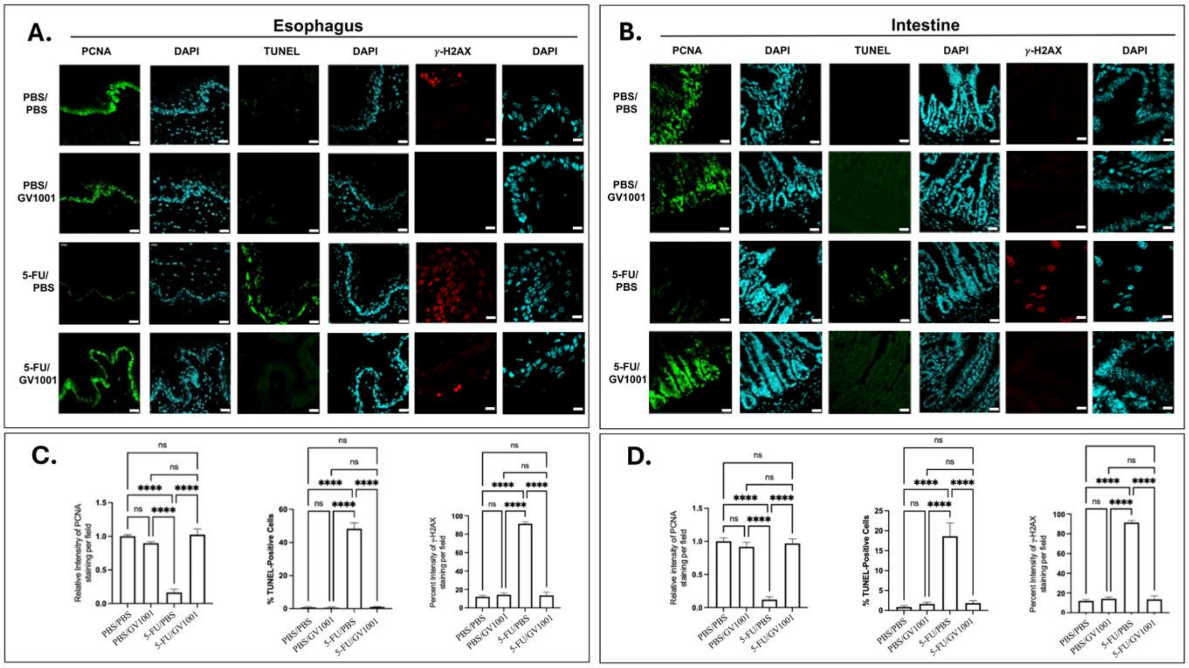

Figure S6. GV1001 mitigates 5-FU–induced apoptosis and DNA damage while preserving proliferative capacity in esophageal and intestinal epithelium. (**A** and **B**) Representative immunofluorescence images of esophageal (**A**) and intestine (**B**) sections from mice treated with PBS/PBS, PBS/GV1001, 5-FU/PBS, or 5-FU/GV1001. Proliferating cells are labelled with PCNA (green); apoptotic nuclei are detected by TUNEL (green); DNA double-strand breaks are marked by γ-H2AX (red). Nuclei are counter-stained with DAPI (cyan). Scale bar, 20 µm. **(C** and **D**) Quantification of PCNA fluorescence intensity per field (left), percentage of TUNEL-positive cells (center), and γ-H2AX fluorescence intensity per field (right), each normalized to the PBS/PBS control group for esophagus (**C**) and intestine (**D**). Statistical significance was indicated: ns = not significant, **** $p < 0.0001$.

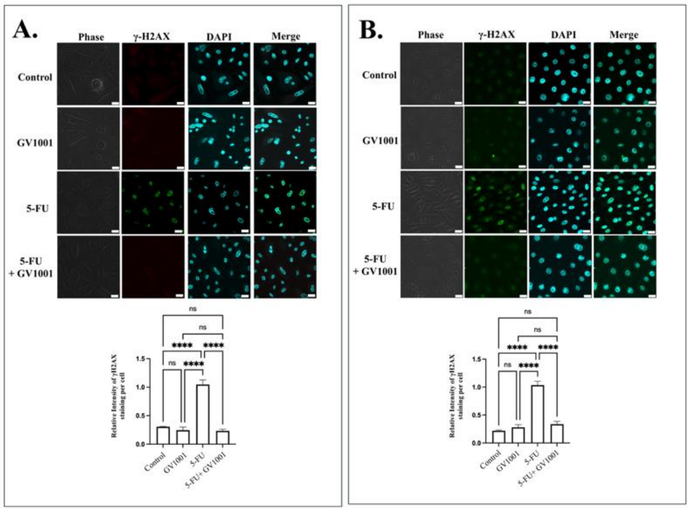

Figure S7. GV1001 reduces 5-FU–induced DNA double-strand-break formation in oral epithelial cell lines. (**A, B**) Representative images of NHOK (**A**) and HOK-16B (**B**) stained for the DNA-damage marker γ-H2AX after treatment with vehicle control, GV1001 alone, 5-FU alone, or 5-FU + GV1001. Phase-contrast, γ-H2AX (green), nuclei are counter-stained with DAPI (cyan), and merged channels are shown. Scale bar: 20 µm. Quantification of TUNEL staining intensity in NHOK and HOK-16B was conducted with ImageJ. Statistical significance is indicated: ns = not significant, **** $p < 0.0001$.

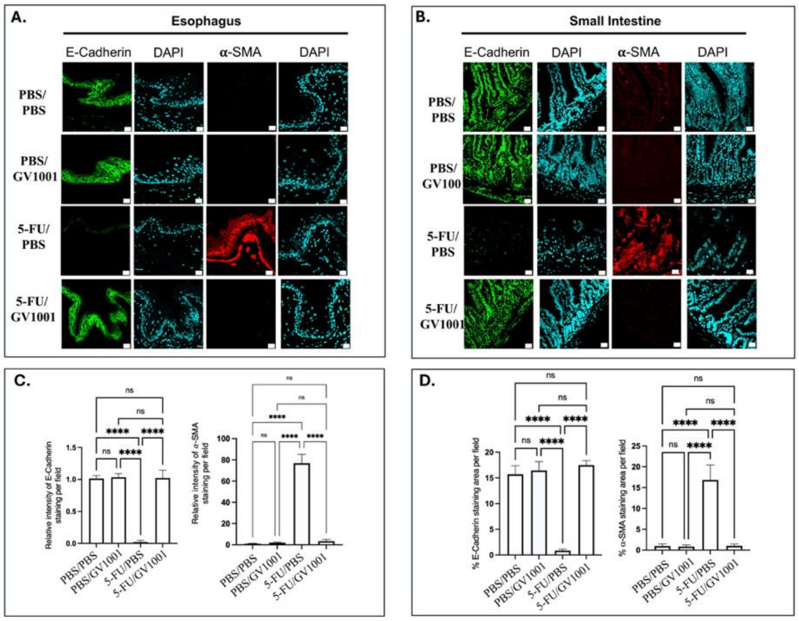

Figure S8. GV1001 preserves epithelial identity and suppresses mesenchymal transition in oral mucosa with 5-FU exposure. Representative immunofluorescence images of the esophagus (**A**) and small intestine (**B**). E-cadherin (green) marks epithelial adherence junctions, and α-SMA (red) identifies mesenchymal-like cells. Nuclei are counterstained with DAPI (cyan). Scale bar: 20 μm. (**C, D**) Quantification of E-cadherin and α-SMA fluorescence intensity in the esophagus (**C**) and small intestine (**D**) using ImageJ. Statistical significance is indicated: ns = not significant, **** $p < 0.0001$.

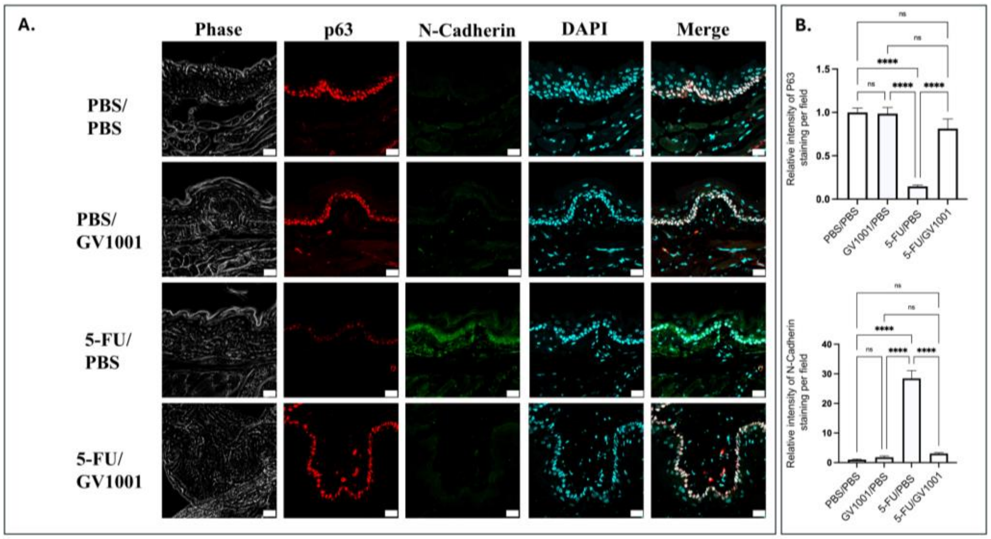

Figure S9. GV1001 preserves the basal progenitor marker p63 and prevents N-cadherin induction in 5-FU–treated esophageal epithelium. (**A**) Representative phase-contrast and immunofluorescence images of esophageal sections from mice receiving PBS/PBS, PBS/GV1001, 5-FU/PBS, or 5-FU/GV100. p63 (red) labels basal keratinocytes, whereas N-cadherin (green) denotes mesenchymal-like cadherin expression; nuclei are counter-stained with DAPI (cyan). Scale bar: 20 µm. (**B**) Quantification of fluorescence intensity per field for p63 (upper graph) and N-cadherin (lower graph), normalized to the mean of the PBS/PBS control group. Statistical significance is indicated: ns = not significant, **** $p < 0.0001$.

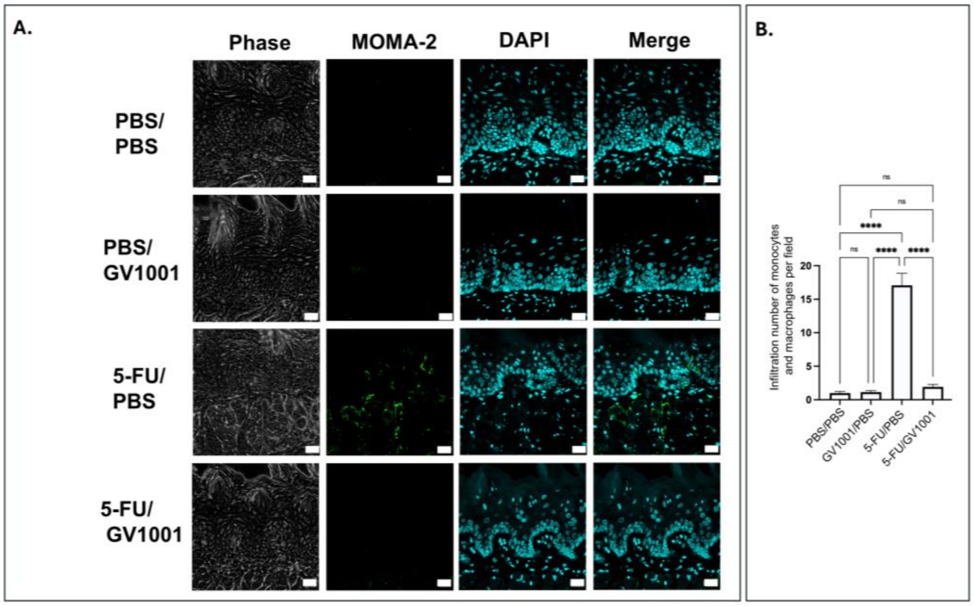

Figure S10. GV1001 limits 5-FU–induced infiltration of monocytes/macrophages in the tongue. (**A**) Representative phase-contrast and immunofluorescence images of tongue sections from mice treated with PBS/PBS, PBS/GV1001, 5-FU/PBS, or 5-FU/GV1001. Infiltrating monocytes/macrophages are labeled with the pan-macrophage marker MOMA-2 (green), nuclei are counter-stained with DAPI (cyan), phase contrast, and merged. Scale bar, 20 µm. (**B**) Quantification of infiltrating MOMA-2–positive cells per microscopic field, normalized to the mean of PBS/PBS control group. Statistical significance is indicated: ns = not significant, **** $p< 0.0001$.

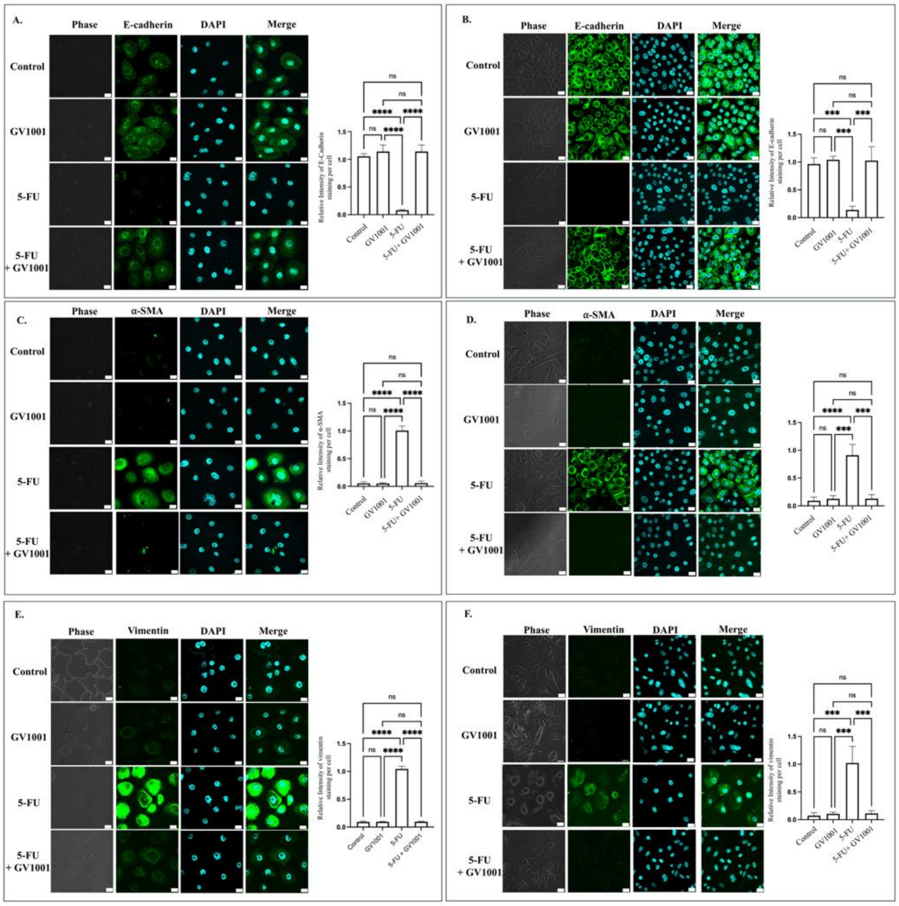

Figure S11. GV1001 counteracts 5-FU–driven epithelial-to-mesenchymal transition (EMT) in normal keratinocytes (NHOK) and HOK-16B cells. (**A–F**) Immunofluorescence analysis of epithelial (E-cadherin) and mesenchymal (α-smooth-muscle-actin, α-SMA; vimentin) markers after 48 h treatment with vehicle (Control), GV1001, 5-FU, or the 5-FU + GV1001 combination. Panels on the left (A, C, E) depict normal oral keratinocytes (HOK-16B); panels on the right (B, D, F) depict NHOK cells. Phase-contrast, marker staining (green), nuclear counterstain (DAPI, cyan), and merged images are shown. Scale bar, 20 µm. Bar graphs adjacent to each panel quantify mean fluorescence intensity per cell for E-cadherin (**A, B**), α-SMA (**C, D**), and vimentin (**E, F**), normalized to the respective mean vehicle control (mean ± SE). Statistical significance was indicated: ns = not significant, * $p < 0.05$, ** $p < 0.01$, *** $p < 0.001$, **** $p < 0.0001$.

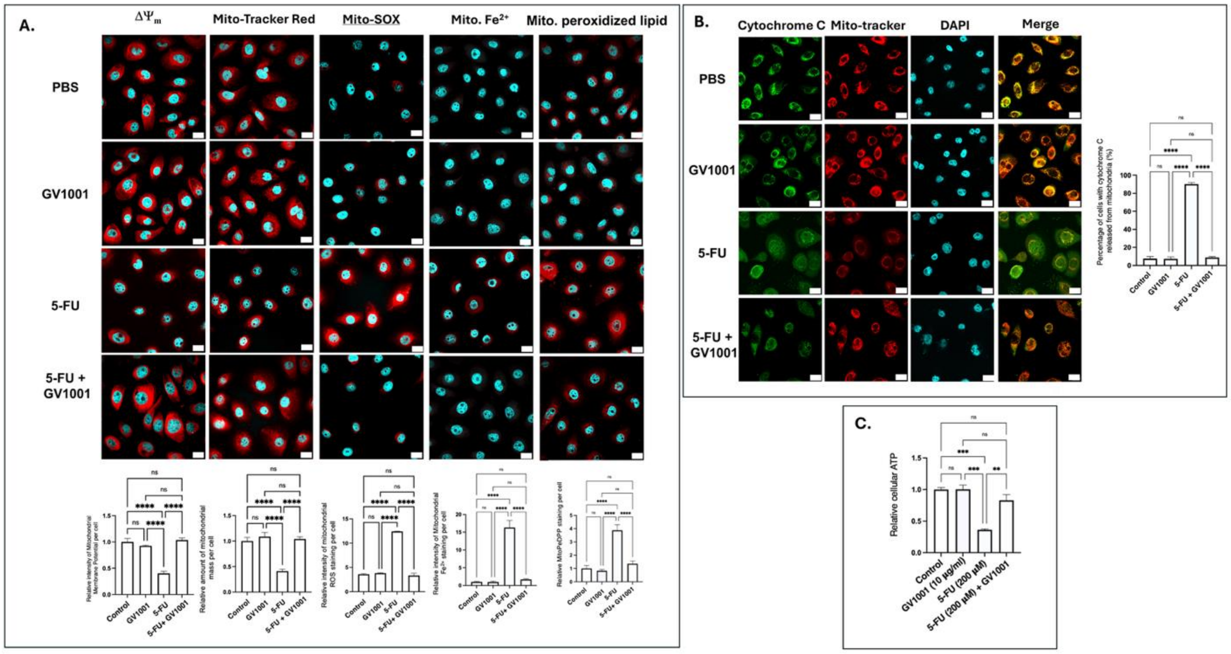

Figure S12. GV1001 preserves mitochondrial integrity and function in 5-FU-treated oral keratinocytes HOK-16B. (**A**) Representative immunofluorescent staining images showing mitochondrial membrane potential ($\Delta\Psi_m$), MitoTracker Red, Mito-SOX (mitochondrial ROS), Mitochondrial Ferrous iron (Fe^{2+}), and mitochondrial peroxidized lipid (MitoPeDPP staining) and DAPI (cyan) in HOK-16B cells. Scale bar: 20 μm. (**B**) Representative immunofluorescent staining images showing cytochrome c (green), MitoTracker Red dye (red), and DAPI (cyan) in HOK-16B cells. Scale bar: 20 μm. (**C**) ATP levels quantified in HOK-16B cells. Significance levels are indicated: ns = not significantly different, * $p < 0.05$, ** $p < 0.01$, *** $p < 0.001$, **** $p < 0.0001$.

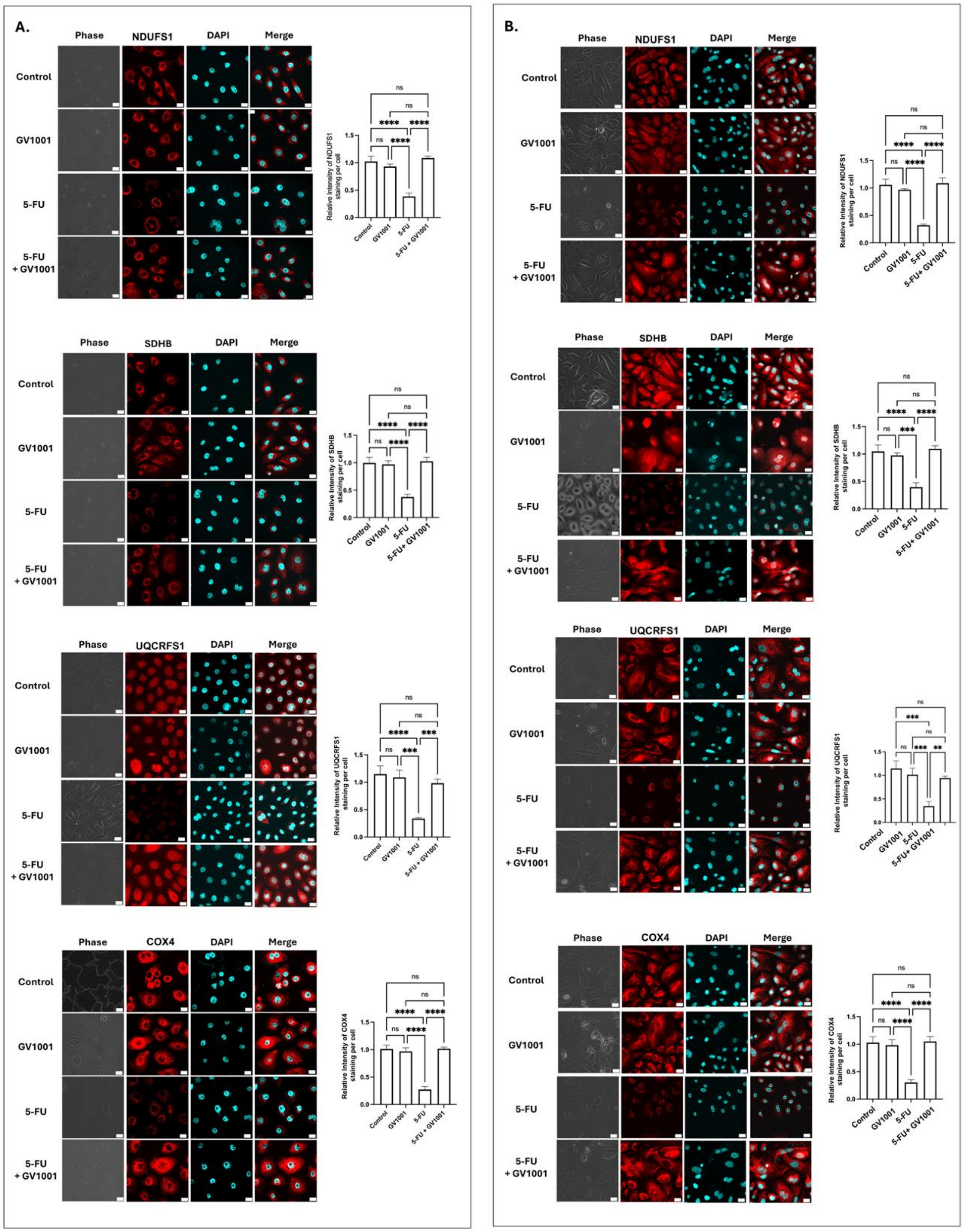

Figure S13. GV1001 preserves mitochondrial respiratory-chain integrity after 5-FU exposure in oral epithelial cell lines. (A-B) HOK-16B (**A**) and HNOK (**B**) cells were treated for 48 hours with vehicle (Control), GV1001, 5-fluorouracil, or the 5-FU + GV1001 combination. Cells were stained for representative subunits of the oxidative-phosphorylation (OXPHOS) complexes: NDUFS1 (complex I), SDHB (complex II), UQCRFS1 (complex III), and COX4 (complex IV). Phase-contrast, protein staining (red), DAPI nuclear counterstain (cyan), and

merged images are shown. Bar graphs adjacent to each image set quantify mean fluorescence intensity per cell for the indicated subunit, normalized to the vehicle control (mean ± SE). Statistical significance was indicated: ns = not significant, * $p < 0.05$, ** $p < 0.01$, *** $p < 0.001$, **** $p < 0.0001$.
